# Examining overlap and homogeneity in ASD, ADHD, and OCD: a data-driven, diagnosis-agnostic approach

**DOI:** 10.1038/s41398-019-0631-2

**Published:** 2019-11-26

**Authors:** Azadeh Kushki, Evdokia Anagnostou, Christopher Hammill, Pierre Duez, Jessica Brian, Alana Iaboni, Russell Schachar, Jennifer Crosbie, Paul Arnold, Jason P. Lerch

**Affiliations:** 1 Autism Research Centre, Bloorview Research Institute, Holland Bloorview Kids Rehabilitation Hospital, Toronto, ON Canada; 2 University of Toronto, Institute of Biomaterial and Biomedical Engineering, Toronto, ON Canada; 3 Department of Paediatrics, University of Toronto, Toronto, ON Canada; 4 Mouse Imaging Centre, The Hospital for Sick Children, Toronto, ON Canada; 5 Bloorview Research Institute, Holland Bloorview Kids Rehabilitation Hospital, Toronto, ON Canada; 6 Department of Psychiatry, University of Toronto, Toronto, ON Canada; 7 Department of Psychiatry, The Hospital for Sick Children, Toronto, ON Canada; 8 Hotchkiss Brain Institute, Departments of Psychiatry & Medical Genetics, University of Calgary, Calgary, AB Canada; 9 Program in Neuroscience and Mental Health, The Hospital for Sick Children, Department of Medical Biophysics, University of Toronto, Toronto, Canada; 10 Wellcome Centre for Integrative Neuroimaging, FMRIB, Nuffield Department of Clinical Neurosciences, University of Oxford, Oxford, UK

**Keywords:** ADHD, Autism spectrum disorders

## Abstract

The validity of diagnostic labels of autism spectrum disorder (ASD), attention-deficit/hyperactivity disorder (ADHD), and obsessive compulsive disorder (OCD) is an open question given the mounting evidence that these categories may not correspond to conditions with distinct etiologies, biologies, or phenotypes. The objective of this study was to determine the agreement between existing diagnostic labels and groups discovered based on a data-driven, diagnosis-agnostic approach integrating cortical neuroanatomy and core-domain phenotype features. A machine learning pipeline, called bagged-multiview clustering, was designed to discover homogeneous subgroups by integrating cortical thickness data and measures of core-domain phenotypic features of ASD, ADHD, and OCD. This study was conducted using data from the Province of Ontario Neurodevelopmental Disorders (POND) Network, a multi-center study in Ontario, Canada. Participants (*n* = 226) included children between the ages of 6 and 18 with a diagnosis of ASD (*n* = 112, median [IQR] age = 11.7[4.8], 21% female), ADHD (*n* = 58, median [IQR] age = 10.2[3.3], 14% female), or OCD (*n* = 34, median [IQR] age = 12.1[4.2], 38% female), as well as typically developing controls (*n* = 22, median [IQR] age = 11.0[3.8], 55% female). The diagnosis-agnostic groups were significantly different than each other in phenotypic characteristics (SCQ: χ^2^(9) = 111.21, *p* < 0.0001; SWAN: χ^2^(9) = 142.44, *p* < 0.0001) as well as cortical thickness in 75 regions of the brain. The analyses revealed disagreement between existing diagnostic labels and the diagnosis-agnostic homogeneous groups (normalized mutual information < 0.20). Our results did not support the validity of existing diagnostic labels of ASD, ADHD, and OCD as distinct entities with respect to phenotype and cortical morphology.

## Introduction

Autism spectrum disorder (ASD), attention-deficit/hyperactivity disorder (ADHD), and obsessive compulsive disorder (OCD) are complex neurodevelopmental disorders. There is emerging evidence that these diagnostic categories may not correspond to conditions with distinct etiology^1–8^, biology^9^, or phenotype^10^, and that they may not represent distinct underlying mechanisms of dysfunction or predict treatment response^11^. In this context, several studies have revealed shared characteristics among ASD, ADHD, and OCD across various levels of analysis (e.g., etiology^1–8^, biology^9,12,13^, and phenotype^10,14–19^), as well as significant comorbidity among these disorders^3,20,21^. These studies commonly rely on case-control designs, which use diagnostic labels to define group-level statistics for comparisons. Although these approaches can identify group differences in means when distributions are close to normal, they cannot characterize group overlap in the presence of large within-group variability that may arise from existence of subgroups within each group. This is an important consideration when analyzing complex disorders, such as ASD, ADHD, and OCD, which present with strikingly large within-disorder heterogeneity in etiology^21–29^, neurobiology^30–36^, and phenotypic presentation^37^.

The between-group overlap and the large within-group heterogeneity motivate a shift away from traditional case-control designs to trans-diagnostic analyses based on diagnosis-agnostic and continuous measures. This approach may provide insight into the structure of individual variability in biology and phenotype, including discovery of homogeneous subgroups and/or continua characterized by different biologies. To this end, we propose a data-driven, diagnosis-agnostic approach to derive sub-groups that share biological and phenotypic characteristics. Previous attempts have been made to discover homogeneous subgroups within each disorder^37–42^ or on a single level of analysis^16,43^, however, to our knowledge cross-disorder, multi-level stratification has not been examined previously.

We examined homogeneity in neuroanatomy, measured by cortical thickness, and core-domain phenotypic characteristics of each disorder. Neuroanatomical similarities can provide an intermediate phenotype that links multiple genetic variants^31^ given that genetic findings are rare and unknown for the majority of individuals with ASD, OCD, and ADHD. Cortical thickness is a heritable measure of cortical columnar structure, suggested to reflect cellular maturational changes in the cortex (i.e., dendritic arborization and pruning, myelination), as well as cognitive and behavioral differences^44–46^.

## Materials and methods

### Participants

Participants were recruited through the Province of Ontario (Canada) Neurodevelopmental Disorders Network (POND), a multi-center research network studying neurodevelopmental disorders. Participants who had capacity to consent provided informed consent. For others, consent was obtained from guardians and assent was obtained from the participants. Ethics approval was obtained from the research ethics boards at Holland Bloorview Kids Rehabilitation Hospital and the Hospital for Sick Children.

The included participants were 6–18 years old, had sufficient English comprehension to complete the testing protocols, and did not have contraindications for MRI. For the clinical groups, a primary diagnosis of ASD, ADHD, or OCD was required. Diagnoses for the clinical groups were confirmed using in-depth assessments (ASD: Autism Diagnostic Observation Schedule–2 (ADOS)^47^ and Autism Diagnostic Interview–Revised (ADI-R)^48^; ADHD: Parent Interview for Child Symptoms (PICS)^49^; OCD: K-SADS and the Children’s Yale–Brown Obsessive Compulsive Scale (CY-BOCS)^50^. The controls did not have a neurodevelopmental, psychiatric and/or neurological diagnosis and were born after 35 weeks gestation.

### Behavioral measures

Our analyses focused on primary domains affected in ASD, ADHD, and OCD, quantified using continuous measures of autism features (Social Communication Questionnaire (SCQ)^51^), inattention (inattentive subscale of the Strengths and Weaknesses of ADHD-symptoms and Normal Behavior (SWAN) rating scale^52^), and obsessive-compulsive traits (Toronto Obsessive Compulsive rating scale (TOCS)^53^). Participants also completed the Child-Behaviour Checklist (CBCL)^59^. Full-scale IQ was estimated using the age-appropriate Wechsler or Stanford-Binet scales.

### Imaging data

Structural MRI data was collected on the 3-Tesla Siemens Trio TIM at the Hospital for Sick Children, in Toronto, Ontario for 184 participants. The remaining participants were scanned after a hardware updated to the Siemens Prisma scanner. Cortical thickness measures were extracted from T1-weighted images using the CIVET pipeline (version 2.1.0)^54^. The pipeline applies a non-uniformity correction on the images^54^ followed by stereotaxic registration to the Montreal Neurologic Institute (MNI ICBM152) template (non-linear 6th generation target)^55,56^. Next, brains were masked, extracted, and classified into gray matter, white matter, and cerebrospinal fluid. Tissue classification images were used to generate gray and white matter surfaces^57–61^. A surface-diffusion kernel was applied^62^, and regions were registered to the automated anatomical labeling atlas^63–65^. Cortical thickness measurements were taken from the distance between the two smoothed surfaces^66^. Quality assurance was carried out at the time of the scan for motion artifact, and was analyzed through the CIVET quality control (QC) analysis pipeline. Scans that were flagged on the QC analysis were manually reviewed for quality and excluded if needed.

Cortical thickness measurements from 76 regions of the brain were regressed against age, sex, and scanner type in a sequential manner and the z-scored residuals were used in subsequent analyses.

### Analysis

**Fig. 1:**
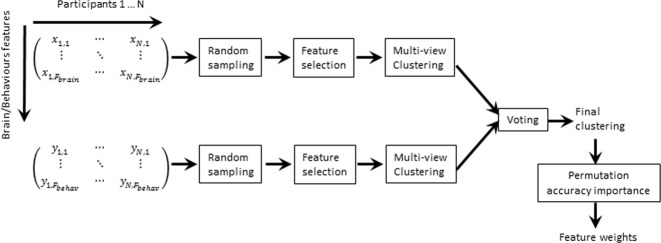
Overview of the analytical pipeline.

Inspired by the concepts of multi-view clustering^67^ and bagging^68^, a machine learning pipeline was designed to analyze the multi-dimensional brain-behavior data. This pipeline (Fig. 1), called bagged-multiview clustering, integrates three features: (1) clustering to discover groups of participants who present with “similar” characteristics in both neuroanatomy and phenotype, (2) bagging to improve cluster stability, and (3) feature weight calculation to determine the cortical regions that contributed most to determining the clusters. The clustering analyses were performed using the Scikit-learn toolbox in Python. Statistical analyses were carried out using R 3.3.3 and Matlab 2017a.

#### Bagged clustering

The bagged-multiview clustering pipeline consisted of bagging and spectral clustering^69^. Resampling methods such as bagging^68^ generate and aggregate decisions based on multiple random subsets of data to improve the accuracy, stability, and generalizability of machine learning algorithms^69^. For this study, a full run of the bagged-multiview clustering pipeline consisted of 50,000 subsamples, each using a random subset of 63.2% of participants, two (of three) dimensions of phenotypic data, cortical thickness measurements from seven (of 76) cortical regions, and the number of clusters randomly chosen between 2 and 15. The size of the random subsets was determined following seminal works in bagging^70,71^. The range for the number of clusters was determined based on visual inspection of affinity matrices. Each iteration generated a participant connectivity matrix, with entries of one if two participants were grouped in the same cluster and zero otherwise. To confirm that the clustering result was indicative of true connections between participants, the analyses were run on two sets created by (1) randomly sampling a uniform distribution across the range of the data, and (2) randomly permuting the cortical thickness and phenotypic data. The distribution of connectivity values due to random chance was computed. Two participants were deemed “similar” if they were grouped together more times than the 99th percentile of the connection values for the random data.

For each iteration of clustering, the following steps were performed. First, using the Gaussian similarity function, affinity matrices were computed for the SCQ, SWAN, and TOCS scores as well as cortical thickness measurements for each of the 76 cortical regions (total of 3 + 76 matrices). The parameter for the Gaussian kernel was set as the 75^th^ percentile of pairwise distances in each measure. Second, a subset of cortex features were chosen for fusion with the phenotype data. The selection maximized within-to-between cluster similarity using sequential-feature-forward selection^72^. Within-cluster similarity was defined as the median of the medians similarities for participants in the same cluster. Between-cluster similarity was the median of the 99th percentile similarity between participants in one clusters and those in all other clusters (99th percentile chosen to deal with the sparsity of the matrix). The overall ratio was computed as the average of the within-to-between ratios, where the ratio for each cluster was weighted by the number of participants in that cluster. Participant similarities were obtained from an affinity matrix resulting from the fusion of cortical thickness and phenotypic matrices. The matrices corresponding to the same data type (cortical measurement or phenotype) were fused using element-wise arithmetic averaging. This type of fusion allows for clusters to match along any of the features combined. This procedure results in two affinity matrices: one for cortical thickness and one for phenotypic measurements. These two matrices were fused using a geometric mean. This requires the final clusters to match across cortical thickness and phenotypic dimensions. To further reduce variability and improve generalizability, the entire pipeline was run 10 times (×50,000 iteration each time) and the median of the participant similarity matrices was used to generate the results reported in the following sections. The 10 iterations were performed further to reduce variability in clustering in a computationally efficient manner.

#### Feature weight calculation

Feature weights were computed based on an adapted version of the permutation accuracy importance^70,71^. In particular, each feature’s prediction accuracy was calculated as the difference between the final labels and the labels generated in iterations where that feature was selected. The values of the feature were then permuted and the accuracy was again calculated. The feature weight was defined as the difference in accuracy before and after the feature is permuted.

#### Agreement between groups

To evaluate the agreement between diagnostic labels and the data-driven cluster assignments, four measures were used:

- Normalized mutual information^73^: Roughly, this measure quantifies that amount of information shared between two clustering assignments. This measure takes on values between 0 (independent clusterings) and 1 (identical clusterings).
- Adjusted Rand score^74^: This measure is based on counting item pairs who fall in the same or different clusters based on two clusterings. The adjusted Rand score ranges between 0 and 1, with 1 indicating perfect agreement between to clusterings.
- Homogeneity^75^: This measure quantifies the extent to which each data-driven cluster contains only participants from a single diagnostic group (0 minimum homogeneity, 1 when each cluster contains only members of a single class).
- Completeness^75^: This measures quantifies how well participants in the same diagnostic group are assigned to the same cluster (0 minimum completeness, 1 perfectly complete assignment).

SCQ, SWAN, and TOCS scores, as well as cortical thickness values were compared across clusters using Kruskal–Wallis tests.

## Results

### Participants

Participant demographic information is shown in Table 1. The diagnostic groups differed significantly in age (χ^2^(3) = 10.1, *p* = 0.02), full-scale IQ (χ^2^(3) = 31.4, *p* < 0.0001), and measures of core-domain symptomatology namely, SCQ (χ^2^(3) = 134.8, *p* < 0.0001), SWAN (χ^2^(3) = 69.5, *p* < 0.0001), and TOCS (χ^2^(3) = 80.2, *p* < 0.0001). The age difference did not survive correction for multiple comparisons. Post-hoc analyses showed that the proportion of male to female participants was higher in the ASD and ADHD groups compared to the OCD and TD groups. Participants in the ASD group had lower median IQ scores compared to the OCD and TD groups, and the ADHD group had lower median IQ compared to the OCD group. Thirty-eight of the 226 participants were missing IQ data.

**Table 1:** Participant demographics.

	ASD (*n* = 112)	ADHD (*n* = 58)	OCD (*n* = 34)	TD (*n* = 22)	Group effect (*p*-value)
Age	11.7 (4.8)	10.2 (3.3)	12.1 (4.2)	11.0 (3.8)	0.02 (ADHD < ASD,OCD)
Sex (f:m)	24:88	8:50	13:21	12:10	0.0004
Full-scale IQ	95.5 (25.5)	98 (23)	120 (18)	110.5 (12.5)	<0.0001 (ASD < OCD,TD; ADHD < OCD)
SCQ	20.5 (10)	6 (7)	4 (4)	1.5 (2)	<0.0001 (ASD > ADHD, OCD, TD; ADHD > TD)
SWAN	5 (5)	6.5 (3)	1 (4)	0 (0)	<0.0001 (ADHD > ASD > OCD > TD)
TOCS	−1 (27.5)	−16.5 (40)	20 (21)	−43.5 (53)	<0.0001 (OCD > ASD > ADHD, TD)

Reported values are median (IQR). P-values are not corrected for multiple comparisons (6 comparisons)

The ASD, ADHD, and OCD groups had significantly elevated scores compared to all groups on their respective core-domain measures (SCQ, SWAN, TOCS; *p* < 0.0001). Interestingly, the ADHD group had significantly higher SCQ scores compared to the TD controls, and the ASD group had significantly elevated SWAN and TOCS scores compared to the TD groups.

In the ASD group, 46 and 40% of the participants met clinical cut-offs on the SWAN and TOCS, respectively. In the ADHD group, 11 and 17% of participants met clinical cut-offs on the SCQ and TOCS. Of the participants in the OCD group, 8 and 24% met the cut-off on the SCQ and SWAN, respectively. None of the TD participants met the cut-offs for SCQ or SWAN, but 2 of the 22 exceeded the cut-off on the TOCS (eTable 1 in the Supplement). The distribution of each of the core-measures scores also evidenced overlap among the diagnostic groups with respect to all three measures (eFig. 1 in the Supplement).

### Cluster-diagnosis agreement

The agreement was <0.2 for the normalized mutual information and adjusted rand scores for cluster numbers ranging from 2 to 14 (perfect agreement corresponds to a value of one). Homogeneity and completeness scores were less than 0.3, indicating that data-driven clusters do not represent a single diagnostic category (eFig. 2 in the Supplement).

**Fig. 2:**
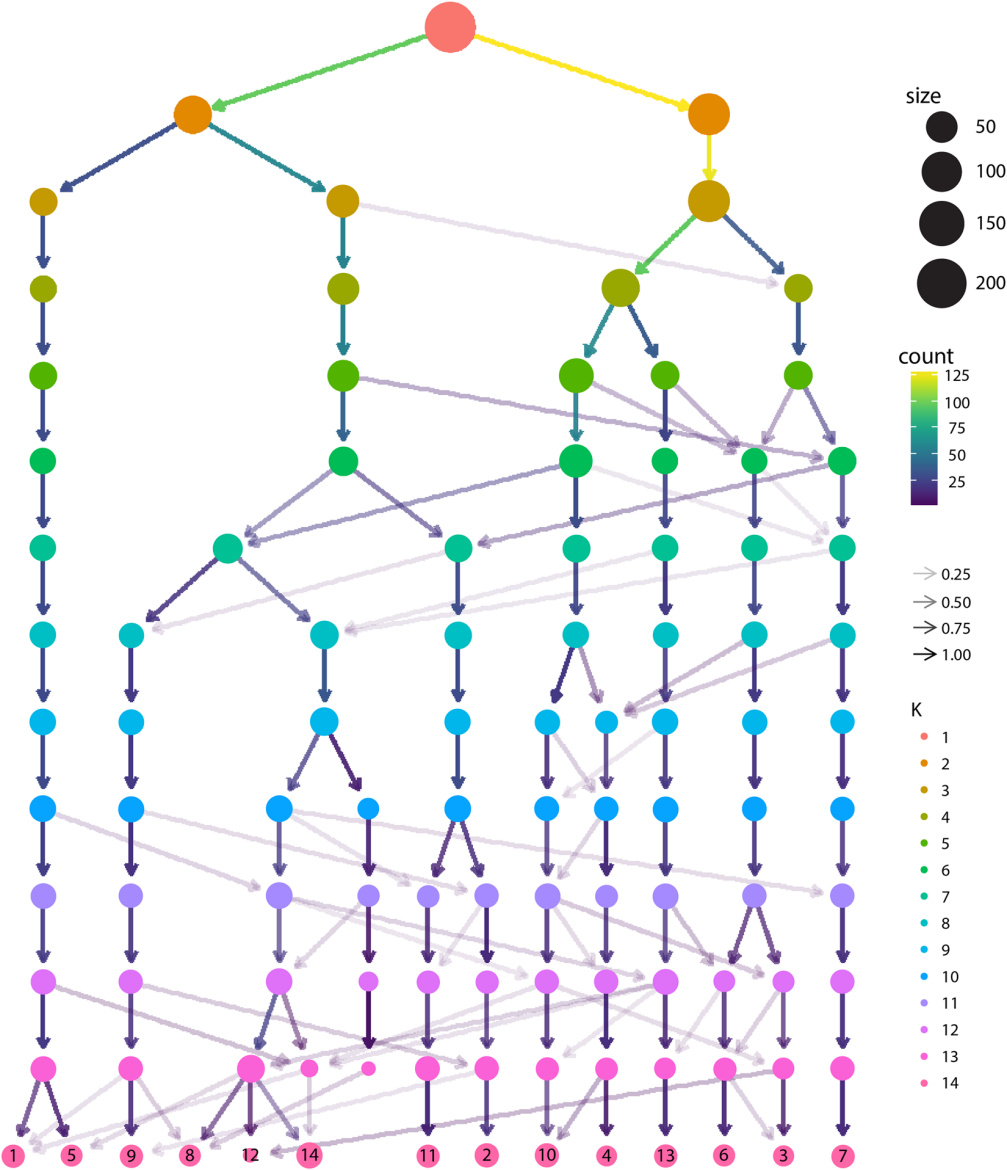
A graphical representation of the emergence of clusters. Different levels in this figure correspond to a different number of clusters used to partition the similarity matrix generated by the bagged-multiview clustering pipeline.

### Clusters

Based on the within-to-between similarity ratio, a 10-cluster solution was chosen for the remaining analyses (eFig. 3 in the Supplement). Figure 2 graphically depicts how clusters emerge as the number of clusters increases. The figure was generated by changing the number of clusters in a spectral clustering algorithm applied to the similarity matrix generated by the bagged-multiview clustering pipeline.

The Kruskal–Wallis test did not show significant cluster differences in age or sex proportions (eFig. 4 in the Supplement). However, the clusters were significantly different in IQ (χ^2^(9) = 23.6, *p* = 0.005). Post-hoc testing showed that cluster 1 had significantly higher mean ranks than clusters 5 and 7 (*p* = 0.03).

### Diagnostic labels

**Fig. 3:**
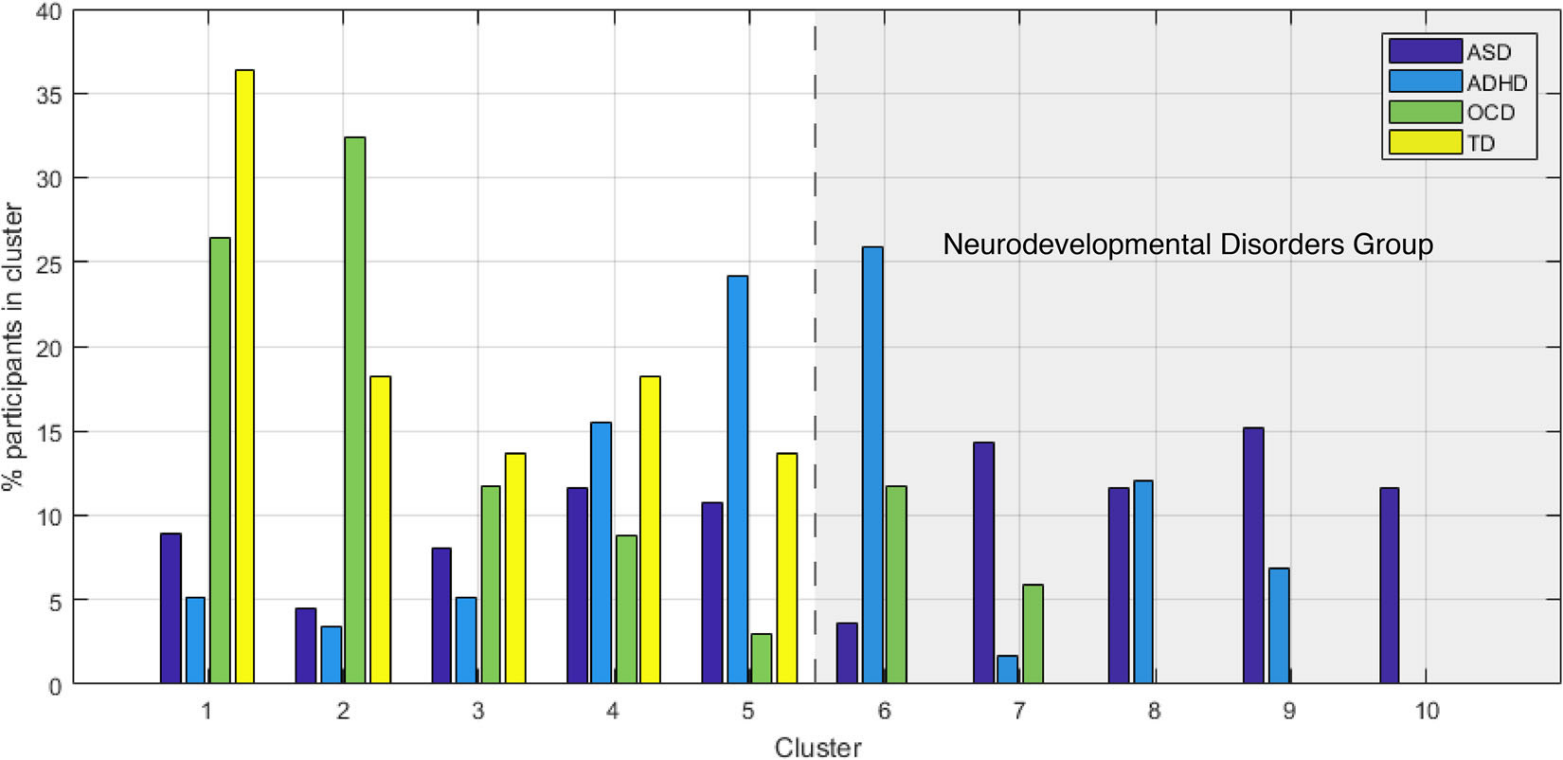
Percentage of participants from the four diagnostic categories in each cluster.

Figure 3 shows the percentage of participants from the four diagnostic categories falling into each of the ten clusters. Most clusters contained participants from multiple diagnostic groups. There was also a group of clusters with participants from the neurodevelopmental groups only (referred to as “neurodevelopmental clusters” from here on). There was also a small cluster of participants with ASD only (cluster 10), containing 12% of the participants with an ASD diagnosis.

The Kruskal–Wallis test revealed a significant difference in SCQ and SWAN scores among clusters (SCQ: χ^2^(9) = 111.21, *p* < 0.0001; SWAN: χ^2^(9) = 142.44, *p* < 0.0001), but the cluster difference in TOCS scores was not significant (eFig. 5 in the Supplement). The clusters were also significantly different in CBCL Social Problems scores (χ^2^(9) = 56.3, *p* < 0.0001) and Attention Problems (χ^2^(9) = 94.8, *p* < 0.0001), but not OCD Problems. Kruskal–Wallis tests showed a significant effect of cluster on cortical thickness in all regions (Bonferroni corrected *p* < 0.002), except for the left lingual gyrus.

**Fig. 4:**
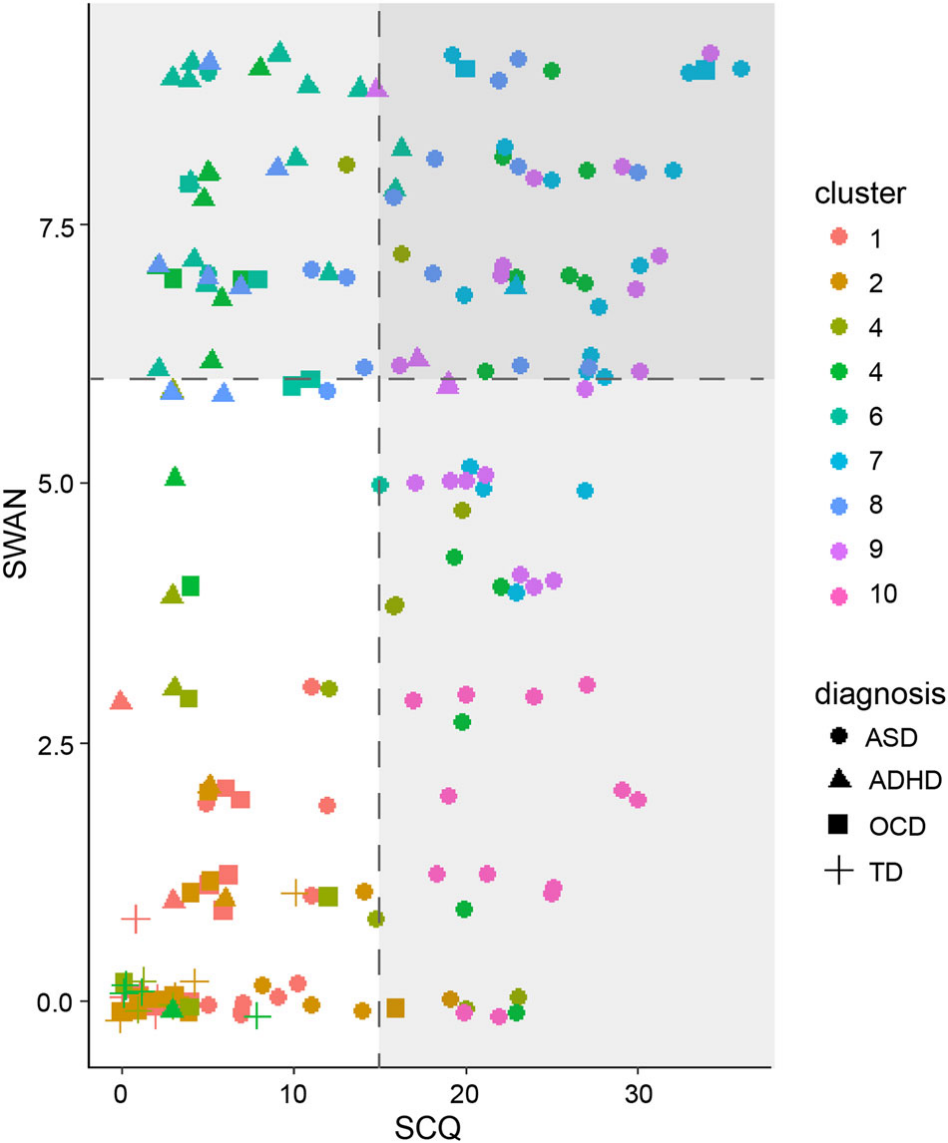
Distribution of SCQ and SWAN score for each data-driven cluster and diagnostic group. Poorly defined cluster (cluster 5) excluded from plots. Values perturbed by random Gaussian noise to enhance visualization.

Figure 4 depicts participant-level SCQ and SWAN scores for the diagnostic groups, as well as the data-driven clusters. This figure highlights the differences between diagnostic classifications and the data-driven solution. The data-driven clusters broadly divide the SCQ-SWAN space into low and high SWAN scores based on a cut-off score of 6; within the low and high SWAN regions, a continuum of SCQ scores can be observed. The neurodevelopmental clusters fall within the high SWAN region, with the exception of the “pure” ASD group.

The participant similarity matrix (eFig. 6 in the Supplement) revealed significant overlap among clusters 1 through 3, and 6 through 9, suggesting a structure more consistent with a continuum rather than distinct clusters within these groups. The matrix also indicates that clusters 5 is poorly defined (low similarity among the participants in the cluster).

### Cortical regions

**Fig. 5:**
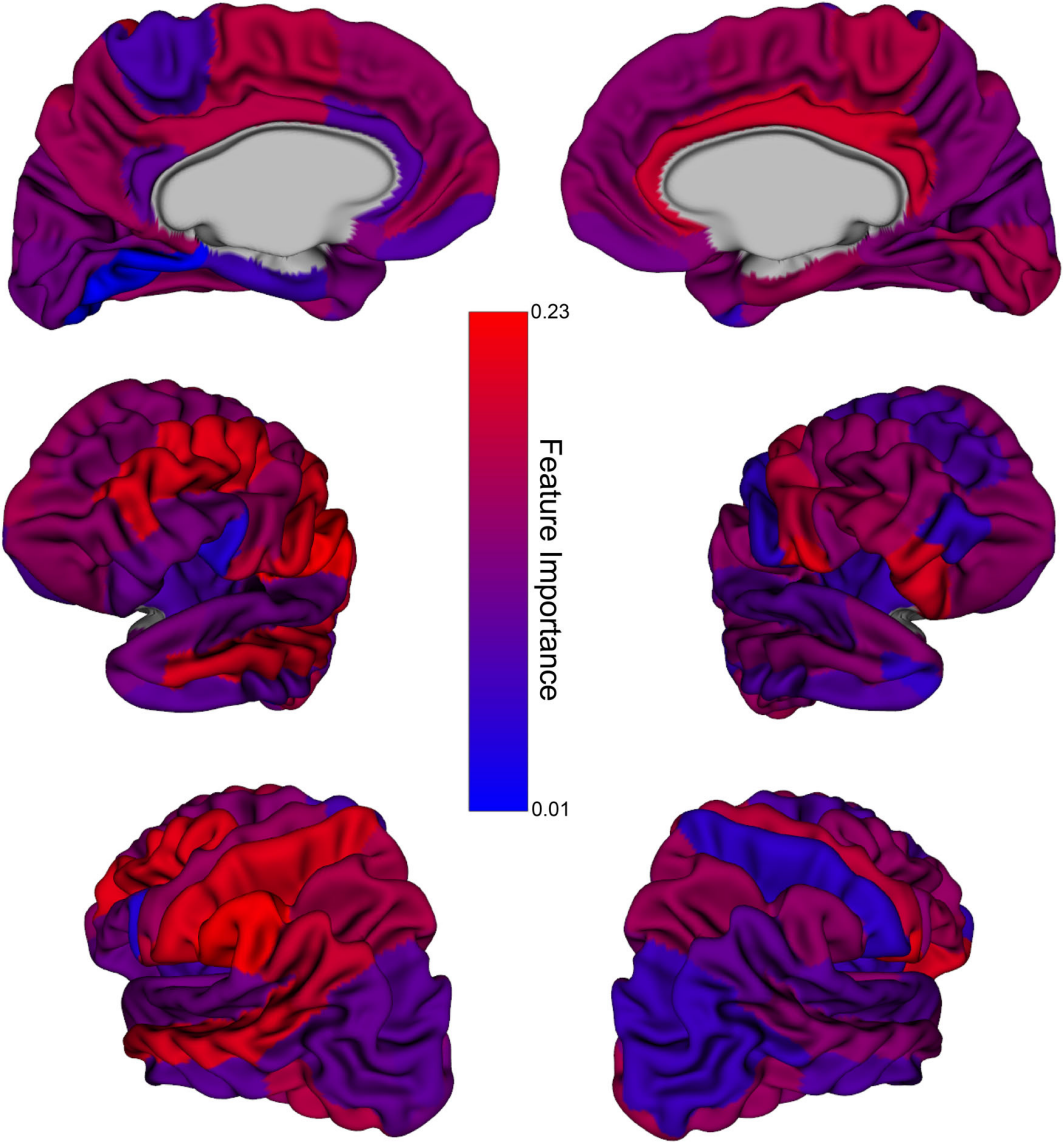
Feature weights associated with each cortical region for cortical thickness data without (left) and with (right) sex correction. Red hues represent higher weights (more contribution to determining the clustering solution).

Figure 5 visualizes the contribution of each cortical region to the clustering solution. The top ten highly weighted features are listed in eTable 2 in the Supplement for reference.

The weight distribution among the regions was relatively uniformly decreasing (eFig. 7 in the Supplement), suggesting that no single region is driving the clustering results.

### Cluster validity

The distribution of connection values used to derive clustering solutions for participant data showed no overlap with the randomly generated data (eFig. 8 in the Supplement).

## Discussion

In this study, we used a data-driven, diagnosis-agnostic approach to examine overlap across three neurodevelopmental disorders (ASD, ADHD, and OCD). Overall, our results suggest that homogeneity in the variables examined in our analyses does not align well with existing diagnostic categories. Instead, we observed that differences in the domains primarily affected in these disorders may exist along a continuum that includes typical development.

### Clusters

We started with a grouping of participants categorized into four diagnostic groups, which differed significantly on scores on SCQ, SWAN, and TOCS. Our analyses resulted in a new grouping of these participants into more homogeneous subgroups, which differed significantly in SCQ and SWAN scores as well as cortical thickness, but did not align well with the original diagnostic labels.

The majority of the data-driven clusters contained participants from multiple diagnostic categories, highlighting shared phenotypes and neurobiologies among the diagnostic groups. Social difficulties and inattention are commonly reported as shared features of ASD, ADHD, and OCD^10,14,20,76,77^. Several studies have also reported shared characteristics in brain structure, function, and connectivity^9,12,78–82^ in these disorders. Our results support the emerging recognition that the existing behaviorally-defined diagnostic labels may not capture etiologically, biologically, and phenomenologically homogeneous groups^29,79,83–89^.

Visually, our results are consistent with the notion that that the ASD-like features, and to some extent inattention traits, exist across a continuum that includes typical development. This model is supported by the substantial etiological overlap between these disorders and typical variation in social communication ability^90^ and inattention^91^. This is also consistent with the notion that multiple susceptibility genetic factors may interact with environmental conditions to lead to a continuous dimension of ASD-like and inattention traits, with neurodevelopmental disorders at the extremes of this continuum^92,93^. This motivates models of neurodevelopmental disorders which focus on continuous variations in traits instead of categorical diagnoses defined based on qualitative cut-offs. Future studies should consider examining other phenotypic characteristics and biological parameters (e.g., metabolic, immune, endocrine markers) to comprehensively describe this continuum.

The data-driven clusters differed significantly in SCQ and SWAN scores, but not TOCS. This pattern was also replicated using the CBCL measures of social, attention, and OCD problems. Moreover, the majority of participants with an OCD diagnosis clustered together with the typical controls. This has been observed in two other studies which examined social perception abilities^10^ and white matter structure^9^ using the same cohort. In addition, a study of a community sample found that those with a sibling with ASD showed more ADHD, but not OCD traits compared to those without a sibling with ASD^20^. Replication on larger samples is needed to further explore shared characteristics and differences across these disorders.

Finally, it is important to note that discovery of the exact clusters/subgroups that can be translated into clinical practice requires replication and integration of findings across a large number of studies and measures. This paper is a first step to accomplish this. Our results motivate a paradigm shift to challenge how ASD, ADHD, and OCD are currently defined, diagnosed, and treated. In particular, this paper adds to the evidence that these diagnoses may not exist as uniquely-defined diagnostic constructs, and highlights the need to discover other groupings that may be more closely aligned with biology and/or response to treatment.

Our results also have implications for the research community. Most existing studies commonly rely on case-control designs, which use diagnostic labels to define group-level statistics for comparisons. These approaches are often not able to characterize group overlap in the presence of large within-group variability that is revealed in our study. In this context, our results highlight the need to move beyond traditional statistical approaches to more advanced computational approaches to examine variability and overlap in/across these disorders. To our knowledge, this is the first examination of cross-disorder, multi-level stratification across ASD, ADHD, and OCD.

### Cortical features

Our results add to the emerging evidence that the existing diagnostic categories may not be associated with unique patterns of difference in brain structure, paralleling a recent study showing significant heterogeneity in brain volume across 26 mouse models of ASD^31^.

Broadly, the regions contributing most to the data-driven groupings were involved in social function, emotion processing, language, attention, and inhibitory control. Many of these regions have been previously implicated in studies of cortical morphology in ASD (e.g., middle temporal gyrus^94–99^, supramarginal gyrus^78,96–99^, angular gyrus^100^, middle frontal gyrus^94,96,99,100^, cingulate^94,97,99^, inferior frontal gyrus^96,98,99^, postcentral gyrus^96,98–100^, inferior temporal gyrus^94,98,99^), ADHD (e.g., cingulate^101–104^, dorsolateral prefrontal cortex^102^, inferior frontal cortex^102^, anterior cingulate cortex^13^, temporoparietal regions^13^), and OCD (e.g., inferior frontal gyrus^105^, anterior cingulate cortex^13,105^, supramarginal gyrus^105^, dorsolateral prefrontal cortex^13^, middle frontal gyrus^12^).

Our results also overlap with those of the very few studies that have examined similarities and differences in brain structure across pairs of ASD, ADHD, and OCD. For example, disorder-specific differences in the left middle temporal gyrus^95^, right supramarginal gyrus^78^, and the prefrontal cortex^106^ have been reported for ASD and ADHD. Looking at ADHD and OCD, differences have been reported in the cingulate cortex and dorsolateral prefrontal cortex^13^. Decreased volume in the anterior cingulate cortex has been suggested as a shared finding in ASD and OCD ^12^.

### Limitations

Our analyses were conducted on a single measure of cortical structure and three phenotypic measures as well as a specific age group. These levels of analyses may not fully capture homogeneity across the disorders. Future work should consider running similar types of analyses using multiple measures that can comprehensively characterize the variability across neurodevelopmental disorders. These include brain structure and function and core and comorbid behavioral domains across the life-span, as well as genetic, epigenetic, metabolic, immune, and endocrine markers.

The sample size used for the analyses reported in this paper was limited, with unequal distribution of participants across the diagnostic groups. Replication with larger sample sizes is needed.

To our knowledge, this is the first study of diagnosis-agnostic homogeneity across ASD, ADHD, and OCD using data-driven discovery. Homogeneity in the variables examined in our analyses did not align well with existing diagnostic categories in the sample studied. These results add to the emerging body of literature questioning the validity of existing diagnostic constructs with respect to having distinct biological and phenotype presentation. The results of this study also highlight the need for a shift from case-control models to more complex analyses that can cope with the large between-disorder overlap and within-disorder variability.

## Supplementary information

Supplementary Material
