# Supplementary material for: Examining overlap and homogeneity in ASD, ADHD, and OCD: a data-driven, diagnosis-agnostic approach
Source: Transl Psychiatry. 2019 Nov 26;9:318. doi: 10.1038/s41398-019-0631-2 (PMC6880188; doi:10.1038/s41398-019-0631-2)
Supplement: Supplementary file 1 — Supplementary Material [file 41398_2019_631_MOESM1_ESM.docx]

# Supplementary Material

*Figure 1. Distribution of core-domain scores for each diagnostic group. Plots are shown for pairs of groups to improve readability.*

*Figure 2. Measures of agreement between diagnostic labels and data-driven clusters versus the number of clusters. Perfect agreement corresponds to a value of one.*

*Figure 3. Within/between similarity ratio versus the number of clusters. The points represent median ratio values over 10 runs of the algorithm. Bars depict interquartile range.*

*Figure 4. Cluster characteristics. Note: 38 participants missing IQ data. For box plots, the central mark depicts the median, the bottom and top edges of the box show the 25th and 75th percentiles, and the whiskers extend to the most extreme data points not considered outliers. The symbol ‘+’ depicts the outliners.*

*Figure 5. SCQ, SWAN, and TOCS scores for each cluster. The central mark depicts the median, the bottom and top edges of the box show the 25th and 75th percentiles, and the whiskers extend to the most extreme data points not considered outliers. The symbol ‘+’ depicts the outliners.*

*Figure 6. Participant connectivity matrix.*

*Figure 7.Features weights versus feature ranks.*

*Figure 8. Distribution of connection values for participant data and randomly generated data.*

*Table 1. Number of participants meeting cut-off for each of the core domain measures.*

*Table 2. Top ten highly weighted features for analysis with and without sex correction.*

*.*

*Figure 1. Distribution of core-domain scores for each diagnostic group. Plots are shown for pairs of groups to improve readability.*

*Figure 2. Measures of agreement between diagnostic labels and data-driven clusters versus the number of clusters. Perfect agreement corresponds to a value of one.*

*Figure 3. Within/between similarity ratio versus the number of clusters. The points represent median ratio values over 10 runs of the algorithm. Bars depict interquartile range.*

Number of participants in each cluster Age of participants in each cluster

Sex proportions Full-scale IQ

*Figure 4. Cluster characteristics. Note: 38 participants missing IQ data. For box plots, the central mark depicts the median, the bottom and top edges of the box show the 25th and 75th percentiles, and the whiskers extend to the most extreme data points not considered outliers. The symbol ‘+’ depicts the outliners.*

 ******

*Figure 5. SCQ, SWAN, and TOCS scores for each cluster. The central mark depicts the median, the bottom and top edges of the box show the 25th and 75th percentiles, and the whiskers extend to the most extreme data points not considered outliers. The symbol ‘+’ depicts the outliners.*

*Figure 6. Participant connectivity matrix.*

**

*Figure 7.Features weights versus feature ranks.*

*Figure 8. Distribution of connection values for participant data and randomly generated data.*

*Table 1. Number of participants meeting cut-off for each of the core domain measures.*

|  | **SCQ (<=15:>15)** | **SWAN Inattention (<6:>=6)** | **TOCS (<=0:>0)** |
| --- | --- | --- | --- |
| **ASD** | 29:83 | 60:52 | 67:45 |
| **ADHD** | 52:6 | 40:18 | 48:10 |
| **OCD** | 31:3 | 26:8 | 3:31 |
| **TD** | 22:0 | 22:0 | 20:2 |

*Table 2. Top ten highly weighted features for analysis with and without sex correction.*

| **Feature rank** | **No sex correction** | **Sex correction** |
| --- | --- | --- |
| 1 | L middle temporal gyrus | L supramarginal gyrus |
| 2 | R inferior temporal gyrus, orbital part | R Rolandic operculum |
| 3 | R Rolandic operculum | L middle frontal gyrus |
| 4 | R middle temporal gyrus | L middle temporal gyrus |
| 5 | R supramarginal gyrus | L postcentral gyrus |
| 6 | L supramarignal gyrus | R inferior frontal gyrus, orbital part |
| 7 | R anterior cingulate and paracingulate gyrus | R median cingulate and paracingulate gyrus |
| 8 | L middle frontal gyrus | R anterior cingulate and paracingulate gyrus |
| 9 | L median cingulate and paracingulate gyrus | L angular gyrus |
| 10 | L Rolandic operculum | L posterior cingulate gyrus |
